# Bilateral Wilms Tumour: Is Neoadjuvant Doxorubicin Necessary?

**DOI:** 10.3390/children12050587

**Published:** 2025-04-30

**Authors:** Tristan Boam, Sri Paran, Israel Fernández-Pineda

**Affiliations:** Department of Paediatric Surgery, Children’s Health Ireland at Crumlin Hospital, D12 N512 Dublin, Ireland; tristan.boam@childrenshealthireland.ie (T.B.);

**Keywords:** Wilms tumour, bilateral, neoadjuvant chemotherapy, doxorubicin

## Abstract

Approximately 5% to 8% of patients with Wilms tumour have bilateral disease. The prevalence of bilateral Wilms tumour (BWT) is higher in individuals with genetic predisposition syndromes than in those without. The goal of therapy is to preserve as much renal tissue as possible without compromising the overall oncological outcomes, utilising neoadjuvant chemotherapy followed by nephron sparing surgery (NSS) if possible. The Children’s Oncology Group (COG) in North America and the International Society of Paediatric Oncology (SIOP) in Europe have developed the main protocols for the treatment of BWT. Both protocols are similar: initial biopsies are not indicated, and they both recommend surgical resection at week 6 or no later than week 12. Chemotherapy includes the use of vincristine and actinomycin-D in both protocols, but the COG approach also includes the use of doxorubicin, which is a cardiotoxic drug with important long-term effects on the cardiac function of childhood cancer survivors. What doxorubicin adds to patients with BWT in terms of radiological tumour response, resectability, long-term renal function and overall survival, is still not very well described and it may be variable depending on the tumour biology. This article describes the current approach for BWT in North America and Europe, focusing on the potential effect that doxorubicin may have on patient outcomes.

## 1. Background

Wilms tumour or nephroblastoma is one of the most common malignant solid tumours presenting in childhood. Residual primitive metanephric cells (nephrogenic rests) are thought to initiate the neoplastic cascade; as common developmental states may exist in both kidneys, it comes as no surprise that 5–8% of Wilms tumours are bilateral [1]. Nephrogenic rests are associated with almost all instances of bilateral synchronous Wilms tumours (BWTs). The condition of persistent nephrogenic rests is sometimes referred to as nephroblastomatosis (NB), and the terminology in the literature varies and has been further subcategorised based on both histological and radiological features. Pre-surgery, it is challenging to distinguish between a Wilms tumour and a proliferating NB lesion, and diseases treated as BWT may in fact turn out to be unilateral or even bilateral NB. Diffuse hyperplastic perilobar nephroblastomatosis (DHPLN) is considered to be the highest risk for malignant transformation, and chemotherapy is usually recommended as the initial treatment [1].

BWTs present particular challenges to both oncologists and surgeons. The need for disease clearance must be weighed-up against long-term morbidity, including the preservation of renal function, and a careful line must be walked between over- and under-treatment.

Globally, there are two differing perspectives on the pre-operative management of BWT, one from the European International Society of Pediatric Oncology Renal Tumour Study Group (SIOP-RTSG), and the other from the North American Children’s Oncology Group Renal Tumours Committee (COG-RTC). Overall, the treatment protocols are similar in that they both classify BWT as Stage V and mandate upfront chemotherapy without histological confirmation [2]. Chemotherapy includes a combination of a mitotic inhibitor, vincristine, and a transcription inhibitor, actinomycin D (VA) to reduce the tumour volume, followed by (ideally) nephron-sparing surgery (NSS) between weeks 6–12.

Core needle sampling is indicated for unilateral tumours with clinical and radiological features in favour of an alternative diagnosis to Wilms tumour, such as renal cell carcinoma and clear cell sarcoma of the kidney, among others. This approach is validated by a study that showed only a 3% misdiagnosis rate after the final histology [3]. Concerns that biopsy may worsen local relapse rates were found to be of only marginal statistical significance in a United Kingdom study, and so far, biopsy does not mandate the upstaging of a tumour under SIOP guidance [4].

For BWT, biopsy at presentation is discouraged, as it is by far the most likely differential diagnosis for a child with bilateral renal masses [5,6]; however, very rare exceptions may include bilateral angiomyolipomata in cases of Tuberous Sclerosis Complex [7]. In addition, BWT is a heterogeneous disease, and it is challenging to obtain samples that are representative of the disease as a whole; pathologists are often unable to differentiate between DHPLN and BWT on core samples alone [8].

The key difference in the protocols is the use of doxorubicin, an anthracycline, by the COG as part of the upfront chemotherapy regimen for BWT. Proponents of its use suggest that it maximises tumour regression and therefore the amount of renal tissue preserved [2]; however, doxorubicin has well-known cardiotoxic properties and its use is associated with worse cardiovascular morbidity and all-cause mortality among long-term survivors [9]. Long-term cardiac effects, as well as pulmonary diffusion defects, have been observed in a St Jude cohort study of 280 Wilms tumour survivors, two-thirds of which were treated with doxorubicin [10]. In a study of approximately 2000 survivors of unilateral, non-syndromic Wilms tumour, treatment with doxorubicin and radiotherapy was associated with significantly higher rates of heart failure, all-cause late mortality, and worse self-reported quality of life compared to those who received VA alone. Patients treated with VA alone also showed no increased risk of adverse outcomes and reported a comparable quality of life to a matched sibling cohort. However, it is possible that adverse cardiovascular effects may be related to radiotherapy or the combination of radiotherapy and doxorubicin. As the treatments are often given in tandem, it is difficult to distinguish [11].

Both SIOP and COG protocols place a high importance on histology as a prognostic indicator.

Typically, Wilms tumours are heterogenous and are characterised histologically by the predominant cell types, which may be blastemal, stromal, epithelial, or anaplastic [2].

Both agree that the presence of either focal or diffuse anaplasia is an adverse factor, but whilst the COG’s pre-chemotherapy classification system is based on the absence (favourable) or presence (unfavourable), and type of anaplasia, the SIOP post-chemotherapy system grades tumours as low-, intermediate- and high-risk, with diffuse anaplasia or blastemal predominance categorised as ‘high risk’. In general, some patients with intermediate-risk and all with high-risk disease will receive a regimen including doxorubicin under the SIOP protocol, as will those with any anaplasia or loss of heterozygosity at chromosomes 1p and 16q and/or stage III+ disease under the COG’s protocol [2].

### 1.1. Historical Wilms Tumour Trials

To successfully treat a child’s cancer only to saddle them with chronic disease in adulthood is an outcome that paediatric oncologists and surgeons strive against, epitomised in the maxim “Cure is not enough”. It has consequently become the objective of trials over the last several decades to lower the treatment burden whilst still preserving good oncological outcomes [12].

One such set of trials was spearheaded by the COG-RTC predecessor, the National Wilms Tumor Study (NWTS) Group. The NWTS I and II trials conducted between 1969 and 1978 showed that the VA combination was effective for disease control, allowing for the avoidance of post-operative radiotherapy and more toxic agents [12,13]. NWTS II saw the randomisation of doxorubicin in addition to VA for more locally advanced or metastatic disease 6 weeks post surgery. Doxorubicin significantly increased the relapse-free survival rate to 77%, compared to 62.5% for VA alone [14]. However, the addition of doxorubicin to VA for stage III tumours was not found to be beneficial in NWTS III [15]. NWTS IV and V focused on reducing the amount of chemotherapy administered, and improvements in the survival rates were evident. The final NWTS V trial further refined the chemotherapy and radiotherapy protocols by adding additional agents such as etoposide and cyclophosphamide for high-risk patients [12,13].

The first of the six SIOP studies began enrolling patients in 1971. SIOP 1 and 2 introduced the concept of using neoadjuvant radiotherapy to reduce the risk of tumour rupture and create a favourable post-operative stage distribution, allowing for reduced post-operative therapy. The third trial, SIOP 5, established that VA pre-operative chemotherapy was equivalent to radiotherapy in terms of outcomes, but spared the patient from long-term effects. SIOP 6, similar to NWTS II, demonstrated a marked benefit in relapse-free survival (74% vs. 49%) when doxorubicin was added to VA for locally advanced disease compared to VA alone. SIOP 9 focused on reducing the duration of chemotherapy, showing that 4 weeks of pre-operative VA was optimal, with no benefit of longer treatment in unilateral disease [16,17].

The UK Wilms Tumour 3 study revisited the question of neoadjuvant chemotherapy vs. primary nephrectomy, trialling the latter vs. 4 weeks of VA for unilateral Wilms tumours. No differences in outcomes were noted between the two treatment arms, but it was hypothesised that pre-operative chemotherapy allows for an assessment of the histological response to treatment, potentially allowing for a reduction in overall chemotherapy [18].

There is a concern that prolonged chemotherapy regimens for presumed bilateral nephroblastomatosis is a risk factor for poor outcomes if the patient later develops BWT. The authors postulated that this may be due to the increased proportion of anaplasia in the eventual resected specimens; however, they do not clarify the type of nephroblastomatosis that prompted the initial treatment [19]. The German Society of Pediatric Oncology and Hematology (GPOH) found that in BWT without metastases at diagnosis, the relapse rates were approximately 15–18% with neoadjuvant chemotherapy for 90 and 120 days; however, this increased to 29% after 120 days and 60% if surgery was delayed by more than 150 days. In the same group, the overall survival (OS) was 91–100% if surgery was performed before 150 days; however, this dropped to 80% in those operated upon after 150 days [20].

### 1.2. Recent Wilms Tumour Trials

The NWTS group was eventually superseded by the COG Renal Tumors Committee and the AREN ‘0’ trials. The first prospective trial for BWT, AREN0534, was launched in 2009, aiming to improve upon the 4-year event-free survival (EFS) rate of 56% for BWT, and the late end-stage renal failure (ESRF) rate of 12% (compared to less than 1% for unilateral Wilms tumours). Due to the difficulty distinguishing between NB and BWT pre-surgery, patients with bilateral renal lesions >1 cm were assumed to have BWT and included. The trial protocol involved the use of neoadjuvant triple-drug chemotherapy (VA + doxorubicin) followed by NSS before week 12, in an attempt to increase the rate of bilateral NSS to 50% and improve EFS to 73%. The trial showed improvements in NWTS-V; the 4-year EFS was 82%. The relapse rate was 12%, and seven of the twenty-three relapsed patients were categorised as diffuse anaplastic histology. Of the 143 patients that received the neoadjuvant triple-drug regimen, 61% required either a unilateral or bilateral nephrectomy, the rest (39%) were able to undergo bilateral NSS [6,21].

The outcomes of BWT from the SIOP WT 2001 study have recently been reported. In this study protocol, non-metastatic BWT were treated with 4 weeks of VA, followed by subjective reassessment for NSS feasibility. Responsive tumours received additional 4-week cycles of VA, before surgery at a timing based on the discretion of the local surgeon (reported as a median of 11.6 weeks (range 2 to 48.8)). In contrast to its counterpart COG study, all patients with bilateral lesions were included, and classified post hoc as either BWT, unilateral Wilms tumour + NB, or bilateral NB. Poorly responsive tumours received doxorubicin or other drugs such as carboplatin or etoposide in addition to VA. Around half of the patients without metastatic disease responded to VA alone, and interestingly, the authors did not find a difference in the percentage of patients that achieved NSS with VA alone or with VA with additional agents. No difference was observed between patients operated on either before or after 12 weeks. At a median follow up of 91 months, the estimated 5-year EFS was 76% and the OS was 88%, whilst the 10-year OS was 84.6% [22].

### 1.3. Effect of Doxorubicin on Renal Functional Outcomes

Causes of ESRF in BWT are multiple; however, early requirement for renal replacement therapy is associated with patients rendered anephric by the necessity of a completion nephrectomy. In addition, BWT patients with tumour predisposition syndromes also have non-cancer-related pathologies such as nephrocalcinosis and focal segmental glomerulosclerosis that heighten the risk of ESRF [23,24].

The opposing viewpoints of the SIOP and COG’s BWT protocols have valid justifications on either side. Bilateral NSS, when compared to nephrectomy in BWT, has been shown to reduce the incidence of ESRF [25]. However, it is also associated with a risk of positive tumour margins. In the event of this or unfavourable histology, irradiation of the remaining renal tissue is mandated, worsening the functional outcomes and increasing the likelihood of ESRF [5].

Proponents of the use of doxorubicin in the neoadjuvant regimen for BWT suggest that it causes a greater reduction in tumour volume compared to VA alone, allowing for more utilisation of bilateral NSS; however, there is a lack of published data supporting this assumption in the case of BWT [2].

Whilst the rate of 39% for bilateral NSS in the AREN0534 fell short of the trial aims, it was considered an improvement, and comparable to the 41% in the SIOP WT 2001 study [6,22].

However, neither study included an in-depth discussion of volumetric tumour response as an independent enabler of bilateral NSS. For BWT, it is not only the reduction in tumour volume that indicates feasibility for NSS, but the location and amount of functional renal tissue. The authors acknowledge that the tumour volume reduction may not be the most important influencing factor on the rate of NSS, and that rather it is the subjective assessment and confidence of the local surgeon, a variable that is difficult to control in any large multicentre study. Indeed, the SIOP WT 2001 study failed to show that the addition of doxorubicin had any influence on the rate of NSS, (although it did demonstrate a better EFS for tumour volumes > 500 mL [5]) and overall, 87% of patients had normal renal function without replacement therapy at a median of 7.4 years [6,22].

### 1.4. Effect of Doxorubicin on EFS

It is challenging to isolate the effects of the addition of doxorubicin on EFS. The SIOP WT 2001 study reported a better EFS in BWT patients treated with VA alone; however, this is likely representative of the positive outcomes associated with chemo-sensitive disease [22]. Conversely, it is difficult to ascribe chemo-resistance alone to worse outcomes, as mature stromal predominant tumours (with a good prognosis) will respond just as poorly to those with diffuse anaplasia [5]. As mentioned in the previous section, SIOP 2001 reported that doxorubicin may be of benefit for unilateral tumours > 500 mL in volume, with a 5-year EFS of 93.3 vs. 66.9 compared to VA alone, although there was not a significant difference in the 5-year OS [5].

The authors of the SIOP WT 2001 report comment on the fact that the COG study only reported a 4-year EFS, as compared to their 10-year follow up, hypothesising that a subgroup of patients may experience long-term effects [6,22]. A follow-up study of the AREN0534 data reported an 8-year EFS of 75%, and revealed that the incidence of distant relapses to the abdomen, lung, and liver were most frequent shortly after diagnosis, within 2 years, and were associated with positive resection margins. Interestingly, negative resection margins and lymph nodes are associated with an increased risk of late relapse to the kidney after 2 years, suggesting that these may be due to the development of second primary tumours [26]. Similarly, in anaplastic BWT, achieving negative margins was found to not significantly improve long-term EFS [27].

Murphy remarks in his commentary on the AREN0534 and SIOP WT 2001 studies that it is likely that a subset of patients were over-treated in the former and under-treated in the latter [28]. It is telling, however, that the bilateral NSS rates and long-term EFS between the studies are comparable, and whilst it is too early to report on late cardiac outcomes from doxorubicin, it is likely based on well-established evidence that patients treated with doxorubicin may experience problems with the AREN0534 protocol.

## 2. Conclusions

Based on the available data, there is currently insufficient evidence to support the doxorubicin intensification of neoadjuvant chemotherapy in BWT. More specifically, there is a lack of data that demonstrates that it (a) reduces tumour volume, enabling more NSS and better renal outcomes and (b) results in better oncological outcomes and EFS. It is imperative that attempts to prevent ESRF with doxorubicin do not consign patients unnecessarily to the severe consequences of cardiac morbidity in later life.

### Future Directions

Alternatives to doxorubicin intensification may represent the best direction for future BWT treatment.

The current SIOP-RTSG Umbrella protocol for renal tumours recommends the avoidance of doxorubicin for poorly responsive BWT, in favour of an alternative strategy involving etoposide, carboplatin, and consideration of a biopsy [5]. Etoposide, like doxorubicin, is a topoisomerase II inhibitor; however, despite the similarities in mechanisms, etoposide is not associated with cardiotoxicity and heart failure in later life [9]. A 1993 study showed the efficacy of etoposide in refractory or relapsed Wilms tumours that had received a variety of previous treatment agents [29]. Carboplatin similarly does not have risks of long-term cardiovascular morbidity; however, it may be associated with renal and ototoxicity [9,30]. A small study demonstrated the feasibility of and marginally better survival from the use of carboplatin monotherapy compared to ActD monotherapy for neoadjuvant chemotherapy [31]. Whilst the previously mentioned difficulties with biopsy in BWT remain, promising alternatives may present themselves in the form of circulating tumour DNA (ctDNA) analysis. Serum and urine samples from children enrolled on the AREN0533 COG trial were analysed with next generation sequencing for ctDNA. The technology was able to detect chromosomal alterations that may be useful in determining the prognosis. Additionally, the detection of ctDNA itself was associated with worse EFS and overall survival compared to those patients with undetectable levels [32]. A major drawback is that of not being able to accurately define a particular histological subtype such as blastemal, epithelial, stromal, etc., with ctDNA, as so far, no correlation has been found. A possible exception to this may be that of the association between detectable TP53 mutations and the anaplastic Wilms tumours [33].

A possible solution may be to combine ctDNA with new imaging methods, such as magnetic resonance diffusion weighting (MRI-DW) techniques like apparent diffusion coefficient. Several studies comparing pre-operative MRI-DW to histology of resected tumours demonstrate its potential value for differentiating stromal and blastemal subtypes from others [34,35].

Another potential avenue to decrease reliance on doxorubicin could be to develop new methods to improve the rates of bilateral NSS. The centralisation of cases and increased surgeon experience are important factors [36], and better reporting of tumour volume reduction and spatial mapping could lead to standardised protocols, in order to more clearly define which cases are suitable for NSS. Technologies such as indocyanine green (ICG) intra-operative fluorescence are already being trialled for NSS in Wilms tumours; normal renal tissue is more ICG-avid compared to tumours, and this technique could potentially allow for greater sparing of normal parenchyma whilst maintaining negative margins [37]. In addition, techniques such as artificial intelligence for tumour and renal parenchyma volume calculation are under investigation [38]. In future, these may be useful for aiding surgeon decision making in NSS.

In summary, the future of the treatment of BWT would benefit from a standardised international protocol encompassing neoadjuvant agents and pre-operative treatment durations, defined criteria for NSS, biopsy, and histological classification, which would be a welcome prospect for the management of this challenging and rare condition.
